# Biographical Sketch of A. E. Petticolas

**Published:** 1871-02

**Authors:** 


### BIOGRAPHICAL.

### BIOGRAPHICAL SKETCH OF A. E. PETTICOLAS.

A. E. Petticolas was born in Richmond, Virginia, in 1824, and died at Williamsburg, Virginia, on the 27th of November, 1868. From an interesting sketch of his life written by Prof. L. S. Joynes, and published in the Richmond and Louisville Journal for February, 1869, we glean the following facts: Dr. Petticolas was educated in the Medical Department of Hampden Sydney College (now the Medical College of Virginia,) where he graduated. He received the gold medal for his thesis on aneurism, and was made Resident Physician of the College Infirmary. In the same year he was appointed Demonstrator of Anatomy, and in the following spring lectured on Materia Medica and Therapeutics in the Summer School of the College. In 1854, by invitation of the Faculty, he delivered the lecture on Anatomy in place of Dr. Johnson, who was unfortunately lost at sea on his return from Europe; and the following Spring was elected full Professor in that branch of medical science. In 1867, he accepted the chair of Anatomy in the New Orleans Medical College, but at the close of the course was compelled to resign on account of bad health. He returned to Richmond and resumed practice, soon after which he received the unsought appointment of Superintendent of the Eastern Lunatic Asylum at Williamsburg. After a brief period of labor in this Institution, death suddenly came, robbing the hospital and the profession at large of an able practitioner, a courteous gentleman, and a good man.

It was his unquestioned merit, continues Prof. Joynes, which alone made for him the successive advancements which marked his professional career, for he had none of the adventitious aids of future or social influence to speed him on the road to success and distinction. His information in all departments of medical science was uncommonly extensive and accurate, and his opinions well matured. As a public teacher, while he could not boast of those brilliant and showy qualities which fall to the lot of a favored few, he was remarkable for the perspicuity, force and effectiveness of his style, and for the thoroughness and fidelity of his instructions. As a surgeon, his judgment and skill commanded the highest respect and confidence of his professional brethren, though his want of worldly tact, his honesty and straightforwardness of character, which made him a stranger to the arts and wiles of self-advancement, and his precarious health, prevented him from attaining that large pecuniary success which would have been the merited reward of his high qualification.—*Transactions of the American Medical Association*, 1870.

